# Elderly people are inherently sensitive to the pharmacological activity of rivaroxaban: *implications for DOAC prescribing*

**DOI:** 10.1007/s11239-020-02326-x

**Published:** 2020-11-01

**Authors:** Emmanouela Kampouraki, Salah Abohelaika, Peter Avery, Tina Biss, Paul Murphy, Hilary Wynne, Farhad Kamali

**Affiliations:** 1 Translational and Clinical Research Institute, Newcastle University, Newcastle upon Tyne, United Kingdom; 2 School of Mathematics, Statistics and Physics, Newcastle University, Newcastle upon Tyne, United Kingdom; 3 Department of Hematology, Newcastle University, Newcastle upon Tyne, United Kingdom; 4 Older People’s Medicine, Freeman Hospital, Newcastle Upon Tyne, United Kingdom; 5 Translational and Clinical Research Institute, Newcastle University, and Newcastle upon Tyne Hospitals, NHS Foundation Trust, NE1 7RU Newcastle upon Tyne, United Kingdom

**Keywords:** Aged, Anticoagulants, Blood coagulation tests, Rivaroxaban

## Abstract

According to both trial and clinical data on direct oral anticoagulants (DOACs) elderly patients are at greatest risk of bleeding. It is unclear whether age *intrinsically* affects anticoagulation response. To investigate the age-related sensitivity to DOACs, we compared the pharmacological activity of the direct factor Xa inhibitor, rivaroxaban, between young and elderly subjects ex-vivo. 36 fit elderly and 30 fit young subjects [median (IQR) age: 83(75–87) vs 30(26–38) years] provided a blood sample. Clotting parameters were measured in the resultant plasma samples incubated with rivaroxaban (100–500 ng/ml). Parametric, non-parametric tests and regression lines adjusted for rivaroxaban concentration and baseline values were used to compare data. Rivaroxaban produced a greater prolongation of both Prothrombin Time (PT) and modified Prothrombin Time (mPT) (both p < 0.001) in the elderly compared to young subjects (with difference in mean PT increasing from 1.6 to 6.1s and for mPT from 23.5 to 71.1s at 100 ng/ml and 500 ng/ml plasma rivaroxaban concentration, respectively). Factor X and factor II activity was significantly lower in the elderly in the presence of rivaroxaban (p < 0.001 for both). Rivaroxaban prolonged time-based parameters and suppressed the amount of thrombin generation to a significantly greater extent in the elderly compared to young subjects [%change from baseline for Endogenous Thrombin Potential (ETP): − 35.0 ± 4.4 vs − 29.8 ± 7.4 nM*min; p = 0.002]. The use of validated DOAC assays will be of considerable benefit for monitoring elderly patients who, because of their increased sensitivity to rivaroxaban, may require lower doses of the drug for therapeutic anticoagulation.

## Highlights

- Elderly patients on DOACs are at highest risk of bleeding complications.
- This study investigated whether age intrinsically affects anticoagulation response to rivaroxaban.
- Elderly subjects are inherently more sensitive to rivaroxaban compared to young subjects.
- Due to age-related increase in sensitivity, elderly patients may require lower rivaroxaban doses.

## Introduction

Direct oral anticoagulants (DOACs), including the factor IIa inhibitor, dabigatran and factor Xa inhibitors, rivaroxaban, apixaban and edoxaban, have been approved for the treatment of thrombosis, the prevention of thromboembolism in orthopedic post-operative patients, and the prevention of thromboembolic strokes in patients with atrial fibrillation (AF); with respect to the latter, DOACs have been demonstrated to be non-inferior to warfarin in clinical effectiveness [1–5]. Prescription numbers for DOACs have grown rapidly both for patients newly diagnosed with AF and for patients previously taking coumarins, in particular those with unstable anticoagulation control [6]. In 2018, they accounted for 31% of treated patients and around 93% of expenditure on anticoagulant therapy in the National Health Service [7].

Bleeding associated with anticoagulation therapy remains a major concern for clinicians and patients. Whilst quantification of the risk of bleeding has been inconsistent due to differences in study sample sizes, patient populations and time frames, studies with DOACs in general indicate an increased risk of gastrointestinal hemorrhage and a lower risk of intracranial bleeding compared to warfarin [8] and with apixaban and edoxaban being superior to rivaroxaban, dabigatran and warfarin in reducing the risk of major bleeding [9].

Subgroup comparisons of trial data between younger (< 75 years) and older (≥ 75 years) patient groups show higher bleeding rates in the older cohort [10], with the most elderly also being at highest risk of bleeding complications in clinical practice [11]. These findings raise the possibility that age intrinsically influences anticoagulation response to DOACs. It is difficult to establish the inherent effect of age on the pharmacological activity of DOACs in vivo, being unable to control for the presence of the potentially confounding clinical characteristics, especially of age-related changes in body mass, decline in renal function, increasing comorbidities and concurrent medication [12]. We therefore set out to determine the effect of age on the pharmacological activity of the direct factor Xa inhibitor, rivaroxaban, by comparing ex-vivo, the effect of rivaroxaban on hematological parameters between a group of community dwelling fit elderly people and a group of fit young people.

## Patients, materials and methods

The study was approved by the Newcastle upon Tyne Ethics Committee (January 2014; REC reference no: 12/NE/0209) and was conducted in compliance with the Declaration of Helsinki.

### Sample size calculation

As there was no *a priori* information available on the extent to which age affects hematological response to rivaroxaban ex-vivo in humans, we performed a power calculation [13] and estimated that a sample size of 30 in each of two groups (30 elderly and 30 young) would be sufficient to detect a clinically significant difference in hematological parameters of 0.7* Standard Deviation (SD) with a power of 80% and alpha of 0.05 based on a *t* test. Therefore, a sample size of 60 subjects was deemed to be sufficient to test the hypothesis that aging enhances the pharmacological activity of rivaroxaban.

### Study subjects

Subjects with liver dysfunction, or other disease that may affect hemostasis and receiving any medication known to affect hemostasis were excluded. Following written informed consent, medically stable (fit) elderly subjects over 65 years were recruited from a local day center and healthy young subjects aged between 18 and 65 years were recruited from amongst university colleagues, who are generally healthy and with no serious underlying conditions. Each subject provided a fasting venous blood sample (20ml) collected in citrated tubes. Following double centrifugation at 2000×*g* for 7 min each, the plasma samples were aliquoted and stored at – 80°C for later analyses. All plasma samples were treated the same way and the storage period was similar between the two subject groups.

### Hematological assessments

All tests were carried out at the accredited Hematology Laboratory at Freeman Hospital, Newcastle Hospitals NHS Foundation Trust. Rivaroxaban pure powder was kindly provided by Bayer Schering Pharma (Berlin, Germany). The powder was solubilized in dimethyl sulfoxide solution (DMSO) according to the manufacturer’s recommendations, first at a concentration of 1mg/ml and then at a final concentration of 10 µg/ml. The proportion of DMSO ranged between 0.01 and 0.05% of the final solution in which coagulation parameters were measured.

On the day of each experiment plasma aliquots were thawed at 37°C for a maximum of 15 min. Plasma samples were incubated with rivaroxaban (0, 100, 250, 400, and 500 ng/ml) at a concentration range similar to that observed in plasma following oral dosing [14]. Control plasma samples were incubated in parallel with the clinical samples to gauge the accuracy of the measurements.

The IL TOP ACL 700 CTS instrument (Instrumentation Laboratory Company, Bedford, MA, USA) was used for all coagulation assays. Prothrombin time (PT), which has been previously shown to strongly correlate with rivaroxaban levels in spiked normal plasma [15], was measured using RecombiPlasTin2G® and activated partial thromboplastin time (aPTT) using HemosIL® (Instrumentation Laboratory Company, Bedford, MA, USA) [16]. Clotting time was also assessed using modified prothrombin time (mPT), which is a PT assay modified by adding calcium chloride (CaCl_2_) to the RecombiPlasTin 2G® reagent to expand sensitivity and enhance assay dynamics. After reconstituting the thromboplastin reagent with distilled water, it was diluted 1:2.25 with 100 mmol/L CaCl_2_ solution, prepared with anhydrous powder (VWR, Pennsylvania, USA) [17]. Clotting factor II (FII) and factor X (FX) activities in plasma were measured with standard clotting assays using respective clotting factor deficient plasma (Instrumentation Laboratory Company, Bedford, MA, USA). The average coefficient of variation for replicate analysis of samples at baseline and with rivaroxaban using control plasma were as follows; for PT (4.9 and 2.9%); for mPT (5.7 and 4.8%); for APTT (6.4 and 4.3%); for FII (7 and 6%); and for FX (13.6 and 12.3%).

FX antigen levels were measured using a commercially available ELISA kit (Diagnostica Stago, Parsippany, NJ, USA). The protocol was performed as described by the manufacturer. The limit of detection for factor X is reported as 0.5%.

### Thrombin generation assay

Thrombin generation assay was performed using the Calibrated Automated Thrombogram with Fluoroskan® Ascent Fluorometer (Thermo Fisher Scientific, Waltham, MA) and the thrombinoscope software (Thrombinoscope BV 5.0). The assay was performed by pipetting 20 μl platelet-poor plasma (PPPH) reagent (20 pmol/L tissue factor) and 10 μl rivaroxaban solution in DMSO (final concentration in plasma: 187.5 ng/ml based on 10 µl of rivaroxaban 1.5 ng/µl stock solution), or 10 μl DMSO (for baseline measurement) into individual wells of a 96-well microtiter plate with 70 μl plasma. After 10 min of pre-incubation at 37 °C, the reaction was started by the addition of 20 μlFluCa-kit. The fluorescence was measured for 120 min at 37°C (excitation, 390 nm; emission, 460 nm). The following parameters of thrombin generation assay were analyzed; lag time, maximum thrombin concentration (peak), time to peak (tt peak), endogenous thrombin potential (ETP) and velocity index.

### Statistical analysis

Data were checked for normal distribution. Where data were not normally distributed, log transformation was performed to achieve normality. Parametric (Student’s *t* test) and non-parametric (Mann–Whitney U) tests were used to compare data; a p value of < 0.05 was considered to be statistically significant. A comparison of regression lines approach (either linear or quadratic models with rivaroxaban concentration) using analysis of covariance was used to compare the pharmacological activity of rivaroxaban between the two groups, additionally adjusting for baseline (by including it as a covariate in the General Linear Model along with rivaroxaban concentration) to allow for differences between patients within groups. Microsoft Excel (Microsoft Corp., Redmond, WA, USA) and Minitab statistical software version 18 were used for data analyses and reporting. Data are presented as mean ± SD unless otherwise stated.

## Results

### Study subjects

Thirty healthy young (10 males) and thirty six fit elderly people (16 males) were recruited into the study between January 2014 and July 2017. The median (IQR) age and weight of the elderly and young subjects were 83 (75–87) vs 30 (26–38) years and 77 (69–89) vs 72 (64–85) kg (p = 0.214, *t* test), respectively. There were more females in the young and elderly groups [20(66.7%) vs 20(55.6%); p = 0.356). None of the subjects in either group had a medical history of liver dysfunction, or other disease that may affect hemostasis, or were receiving any medication that affects hemostasis.

### Hematological measurements

**Fig. 1:**
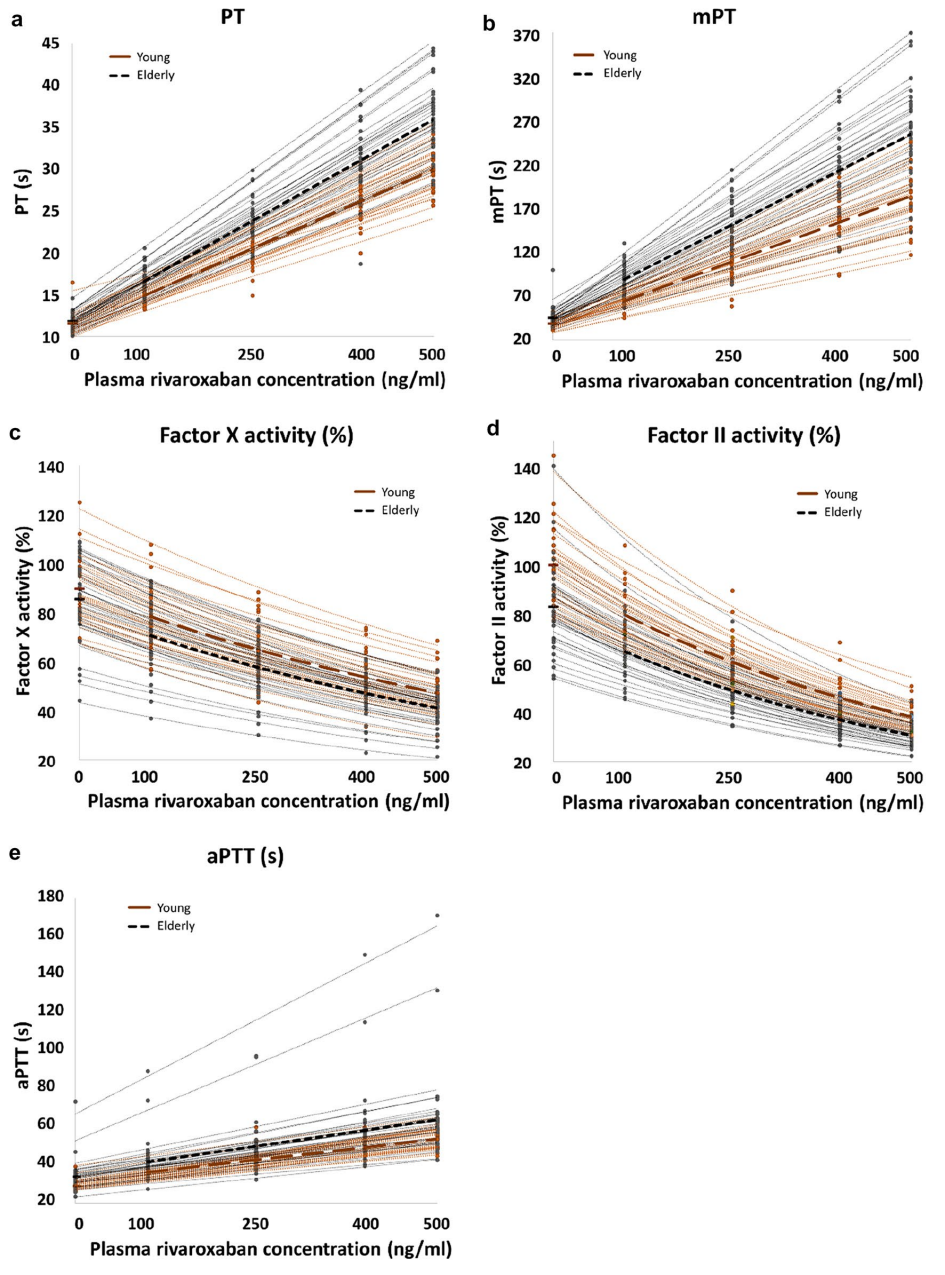
Individual data points and mean PT (**a**), mPT (**b**) prolongation, FX axtivity (**c**) and FII activity (**d**) inhibition and aPTT (**e**) prolongation by rivaroxaban per subject group. Means are presented in bold hashed lines

Elderly subjects had a significantly longer PT and mPT at baseline (p = 0.022 and p < 0.001 respectively; *t* test, Table 1). Both PT and mPT were linearly prolonged with increasing rivaroxaban concentration (Fig. 1a and b). Rivaroxaban caused a significantly greater prolongation of PT and mPT (p < 0.001 for both) in elderly subjects compared to young subjects after adjusting for baseline PT and mPT respectively. The difference in mean PT and mPT between the two groups increased with increasing concentration of rivaroxaban, ranging from 1.6 to 6.1s for PT and 23.5 to 71.1s for mPT (Table 1, Fig. 1a and b).

**Table 1:** Hematological parameters at baseline and in the presence of rivaroxaban and statistical test results for the two subject groups

Rivaroxaban concentration (ng/ml)	*Mean (SD)*		*% change from baseline*		*t* test p value	Regression models	
	Young	Elderly	Young	Elderly		p value	R-sq (%)
PT (s)							
0	11.6 (1.1)	11.9 (0.9)			0.022	< 0.001	90.76
100	15.1 (1.2)	16.7 (1.6)	32.4	39.9			
250	20.2 (1.9)	23.8 (2.9)	75.6	99.6			
400	26.2 (2.6)	30.9 (4.5)	130.6	161.9			
500	30.0 (2.5)	36.1 (4.5)	160.3	202.7			
mPT (s)							
0	38.7 (4.4)	45.4 (10.5)			< 0.001	< 0.001	79.86
100	65.4 (9.5)	88.9 (17.9)	70.1	102.1			
250	105.6 (21.0)	151.1 (32.5)	173.7	244.0			
400	155.9 (31.7)	212.6 (47.0)	305.0	385.5			
500	182.9 (36.6)	253.9 (55.7)	374.6	479.4			
FII activity (%)							
0	99.7 (15.3)	82.7 (17.8)			0.002	< 0.001	94.67
100	79.1 (10.8)	64.5 (10.6)	− 19.6	− 20.2			
250	60.9 (9.3)	49.2 (8.8)	− 34.9	− 39.9			
400	45.7 (7.3)	36.5 (5.2)	− 53.6	− 54.4			
500	38.3 (4.8)	30.9 (4.8)	− 58.9	− 62.0			
FX activity (%)							
0	89.7 (13.5)	85.5 (16.6)			0.281	< 0.001	94.99
100	78.5 (13.2)	71.0 (13.8)	− 12.8	− 16.5			
250	64.5 (11.7)	57.6 (11.5)	− 28.5	− 32.8			
400	53.6 (10.3)	46.4 (9.5)	− 43.1	− 45.5			
500	47.3 (8.8)	41.7 (8.7)	− 47.7	− 51.2			
aPTT (s)							
0	29.0 (2.6)	33.9 (7.9)			< 0.001	0.03	84.22
100	36.5 (3.0)	42.1 (11.0)	27.3	24.2			
250	42.8 (5.0)	49.5 (13.3)	47.8	46.1			
400	49.5 (5.3)	58.8 (20.2)	73.1	72.5			
500	53.8 (5.4)	63.8 (23.5)	85.8	86.4

Mean baseline FX activity was not different between the young and elderly subjects (89.7 ± 13.5% v 85.5 ± 16.6% respectively; p = 0.281, *t* test, Fig. 1c). FX activity was suppressed non-linearly and was significantly lower in the elderly than young subjects across the rivaroxaban concentration range (p < 0.001), with the difference in mean FX activity between the two groups ranging from 5.6 to 7.6%. However, the extent of inhibition, as demonstrated by the rate of decline in FX activity, was similar between the two groups (Table 1). According to the ELISA assay, functional FX antigen levels were lower in the elderly compared to the young subjects (mean ± SD: 70.4 ± 16.3% vs 83.3 ± 13.4%, p = 0.001, *t* test). Factor X specific activity (FX activity/antigen levels ratio) was significantly greater in the elderly compared to the young subjects [median (IQR): 1.21 (1.09–1.40) v 1.05 (1.02–1.12)] respectively, p = 0.005; Mann–Whitney U test].

At baseline, the elderly subjects had a significantly lower mean FII activity, by 17%, compared to young subjects (p = 0.002, Table 1). Factor II activity was suppressed in the presence of rivaroxaban in a concentration-dependent, non-linear manner (Fig. 1d). Factor II activity, adjusted for baseline, was significantly lower in the elderly group compared to the young group across the entire rivaroxaban concentration range studied (p < 0.001) with the difference in mean FII activity ranging from 7.4 to 14.6%. The rate of decline in factor II activity with increasing rivaroxaban concentration was slightly greater in the young subjects compared to the elderly subjects, owing to the young subjects having a higher baseline FII activity.

Baseline aPTT was longer in the elderly compared to young subjects (p < 0.001; *t* test). In the presence of rivaroxaban aPTT was prolonged in a concentration-dependent, linear manner in both groups; however, after adjusting for baseline aPTT prolongation was greater in the elderly compared to young subjects across the entire rivaroxaban concentration range studied (p = 0.03, Table 1, Fig. 1e).

### Thrombin generation assay

At baseline, the elderly subjects had a longer initiation phase of thrombin generation, as demonstrated by the longer lag time (p = 0.01) and time to peak (p = 0.001), and significantly lower ETP (p = 0.004), peak thrombin concentration (p < 0.001) and velocity index (p < 0.001) compared to the young subjects (Table 2).

**Table 2:** Thrombin generation assay parameters, at baseline and with the presence of rivaroxaban (mean ± SEM)

		Lag time (min)	Peak (nM)	ttpeak* (min)	ETP^∂^ (nM*min)	Velocity index (nM/min)
Baseline	*Young*	2.94 (0.07)	322.88 (8.23)	5.54 (0.11)	2396.90 (57.01)	127.71 (5.41)
	*Elderly*	3.31 (0.13)	263.93 (7.93)	6.43 (0.19)	1978.4 (63.87)	87.66 (4.78)
p value		0012	0004	< 0.001	0001	< 0.001
Rivaroxaban^^^	*Young*	9.60 (0.21)	66.98 (4.15)	25.56 (0.74)	1682.16 (51.60)	4.75 (0.58)
	*Elderly*	11.58 (0.64)	41.34 (1.78)	29.12 (0.86)	1279.71 (38.32)	2.56 (0.24)
p value		0007	< 0.001	< 0.001	0003	0001

*ttpeak time to peak

^∂^*ETP* endogenous thrombin potential

^^^187.5 ng/ml final concentration

**Fig. 2:**
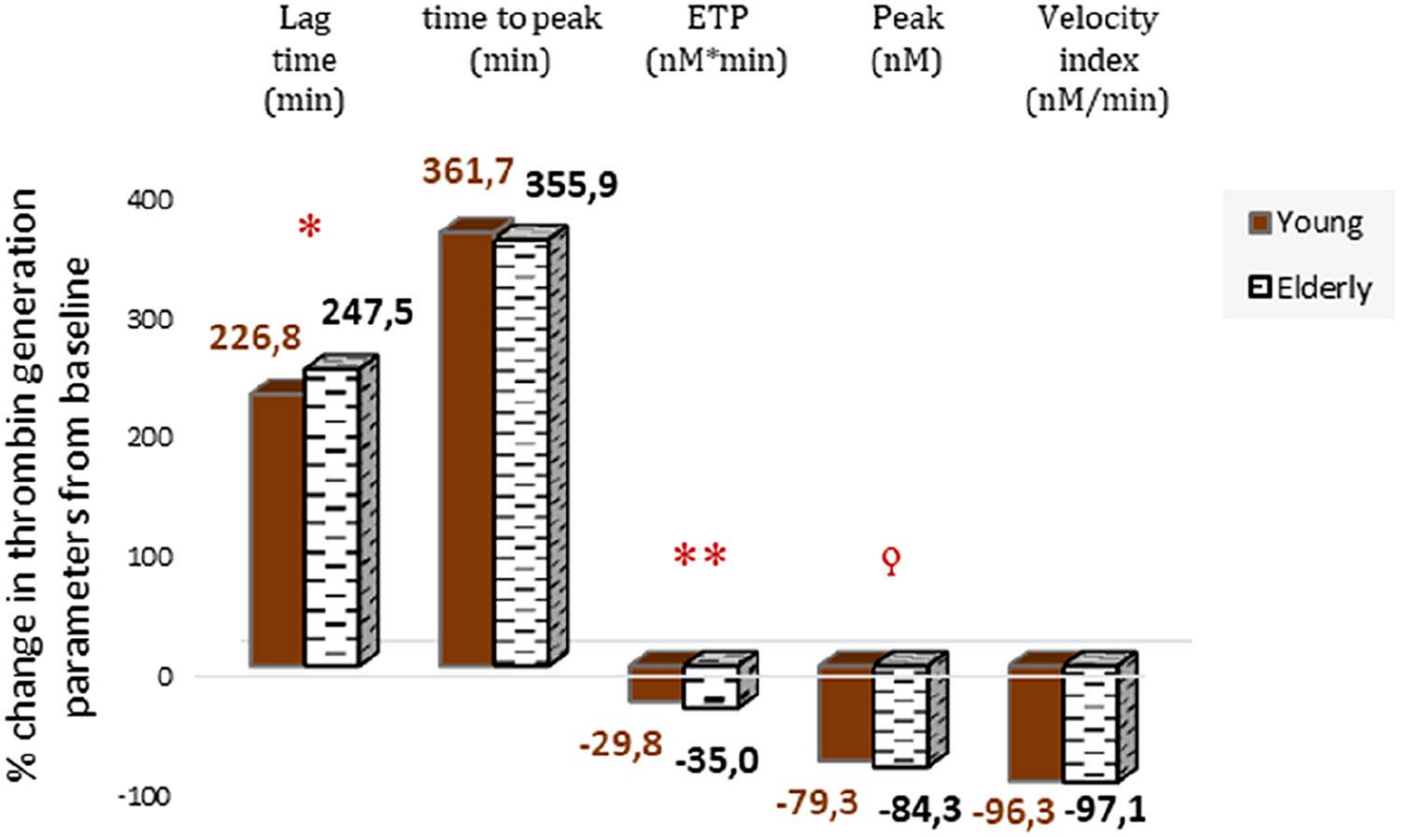
Change from baseline in thrombin generation assay parameters. *p < 0.05 (*t* test), **p ≤ 0.01 (*t* test), ^ϙ^p ≤ 0.01 (Mann–Whitney test)

In both young and elderly subjects rivaroxaban (187.5ng/ml in plasma) significantly affected the key parameters of the thrombin generation assay (Table 2). Rivaroxaban caused a greater prolongation of lag time as demonstrated by the greater percentage difference from baseline in the elderly compared to the young subjects (p = 0.03). Rivaroxaban also suppressed the propagation phase of thrombin generation to a greater extent in elderly subjects compared to the young, as shown by a greater decrease from baseline in both peak (p < 0.001) and ETP (p = 0.002). The change from baseline for both time to peak and velocity index were comparable between the two groups (Fig. 2).

## Discussion

Rates of bleeding are higher in older than in younger patients, whether in the trial situation or in the real-world. The rates of major bleeding in the ROCKET AF study, which compared rivaroxaban to warfarin for efficacy and safety, were 4.63 per 100 patient-years in those aged 75 years or older and 2.74 in younger patients taking rivaroxaban [18]. The incidence rate of hospitalization for DOAC-related bleeding has been reported as 3.44 per 100 patient-years (95% CI 2.35–4.86) with 87% of the admissions being aged ≥75 years, reflecting both the age profile of those prescribed these drugs and their increased risk of bleeding due to age, renal insufficiency, comorbidities and concomitant medication. Of these patients, 19% were receiving the maximum therapeutic daily doses in spite of dose reduction recommendations [19]. Elderly people were not well represented in the major clinical trials evaluating the safety and efficacy of DOACs and this is of concern for clinicians prescribing these agents. In the ROCKET-AF trial, for example, the median age of the patients on rivaroxaban therapy was 73 years and only a quarter of the patients were 78 years of age or older [2]. Post-marketing surveillance studies have reported bleeding rates associated with rivaroxaban to be similar to those reported in the ROCKET-AF trial (2.86 per 100 person-years (95% CI 2.61–3.13) and 3.6 per 100 person-years (HR 1.04 (95% CI 0.90–1.20) respectively) [20–24]. However, a real-world observational study reported a higher bleeding event rate of 17.2% for rivaroxaban compared to 7% for dabigatran, and 8.7% for apixaban [25].

Secondary analysis of the randomized controlled trials [1–4] indicate that, as might be expected, higher drug levels or anticoagulant effects are associated with higher bleeding rates, and lower levels with higher thromboembolic stroke rates. Age-related pharmacokinetic changes contribute to the age-related increase in bleeding rates with anticoagulants. A study of rivaroxaban pharmacokinetics in healthy subjects, for example, noted that the area under the concentration-time curve of rivaroxaban was 41% higher in those over 75 years than in those aged 18–45 years after 10mg rivaroxaban, and inhibition of factor Xa activity and prolongation of prothrombin time were also higher. These changes were attributed to reduced rivaroxaban clearance mainly due to decreased renal function, and the influence of age was not considered clinically relevant, although cannot be ruled out by these data [26]. Current guidelines recommend dose adjustment for DOACs in relation to serum creatinine levels on the basis that renal impairment increases systemic drug exposure; a meta-analysis of randomized trials indicates that, in patients with renal insufficiency, the recommended doses of DOACs are non-inferior and relatively safe compared to warfarin [27].

The evaluation of the intrinsic pharmacological effect of age on the anticoagulation response to DOACs in vivo is challenging, being unable to separately determine the effect of the individual co-factors which influence drug exposure, which include declining renal function, change in body mass, concomitant medication and illness. To overcome this, we compared the pharmacological activity of rivaroxaban in groups of fit elderly and young subjects, ex-vivo. Of the elderly subjects in this study, 72% were aged 78 years and over, representative of the age in the elderly population with AF who are prescribed anticoagulation therapy. None of the study subjects used any drugs which affect hemostasis or interact with rivaroxaban’s mode of action.

Rivaroxaban concentrations chosen for this study were in the range of the plasma rivaroxaban concentrations reported following therapeutic dosing, which are sufficient to cause almost complete inhibition of thrombin generation [14, 28]. Rivaroxaban is a selective, reversible, direct factor Xa inhibitor, which inhibits clot-bound factor Xa as well as prothrombinase activity, therefore prolonging clotting times. It inhibits thrombin production and thus the amplification processes of coagulation through the inhibition of endogenous factor Xa. We measured PT, the time taken to initiate clot formation after the stimulation of the extrinsic coagulation pathway and factors common to both systems. Of all the DOACs, PT is the clotting parameter most responsive to rivaroxaban, and can be used to detect the presence of this drug in plasma. We also measured aPTT which quantifies the integrity of the intrinsic system and the common components, the thrombin generation assay which provides an overall evaluation of coagulation by measuring not only the time to initiate thrombin generation but also the amount of thrombin formed after stimulation with tissue factor, and FII and FX clotting protein activities. Previous studies have shown a very close correlation between rivaroxaban concentration and the inhibition of factor Xa activity and prolongation of PT (R^2^ = 0.99–1.00) and aPTT for spiked normal plasma [14, 15, 29]. The large variability in results for clotting tests between different studies which has been observed depending on the reagents used [15], will not have affected our results, as the same reagents were used to test all the samples.

Rivaroxaban produced a greater prolongation of prothrombin time (both PT and mPT) in the elderly than in the young subjects. There is evidence that the coagulation system becomes more active with increasing age, while fibrinolysis is impaired. Physiological changes taking place with increasing age can affect the production of functional clotting proteins [30–34]. We found that FX specific activity was higher in the elderly compared to the young subjects due to the elderly subjects having a similar baseline FX activity to young subjects but with a lower amount of protein expression. Rivaroxaban inhibited FX activity to a similar extent in both groups. However, the resultant FX activity was lower in the elderly compared to young subjects across the rivaroxaban concentration range studied which signifies that rivaroxaban has a greater net pharmacological effect in the elderly. Baseline FII activity was found to be significantly lower in the elderly compared to young subjects and rivaroxaban suppressed FII activity in a similar pattern to that of FX activity. Rivaroxaban prolonged time-based parameters of thrombin generation (lag time) and suppressed the rate and amount of thrombin generation (peak thrombin and ETP) to a significantly greater extent in elderly subjects compared to young ones. These results support the hypothesis that elderly subjects are more sensitive to the pharmacological activity of rivaroxaban and that age solely could be an important pharmacodynamic contributor to the reported increased incidence of bleeding associated with DOAC therapy in older patients [35].

Accurate, validated and clinically approved assays for use with DOACs have been developed and, when rapid results are more widely available, it is anticipated that they will be of considerable benefit, for example in patients requiring invasive procedures, and those with bleeding or thromboembolic events to assess over and under anticoagulation. More routine use of these assays before bleeding occurs might also be of benefit to vulnerable groups such as those with renal impairment and elderly patients who, because of their increased sensitivity to rivaroxaban, and potentially other DOACs yet to be investigated, may require lower doses of the drug to achieve therapeutic anticoagulation. Clinical experience with DOACs is too short to provide models of use which can take into account the influences of renal function, comorbidities, medication, weight and age to minimize the complication of bleeding and optimize the benefits of therapy. Whilst therapeutic outcomes observed in randomized trials support the use of fixed doses of DOACs [5], monitoring has the potential to make a contribution to improving the clinical effectiveness of these already valuable drugs. We recommend that monitoring can help address the issue of increased sensitivity of older patients. Currently, there is no consensus regarding optimal concentration ranges for therapeutic anticoagulation with rivaroxaban and data are limited to phase 3 clinical trials and some small-scale real-world studies. When plasma rivaroxaban concentration ranges for therapeutic anticoagulation with rivaroxaban are established through further research, can we further investigate the variation in plasma drug levels among older patients and its consequences in relation to bleeding and thrombotic episodes.

Our results are based on coagulation tests performed ex vivo with plasma from volunteers which was spiked with rivaroxaban. Prospective studies in patients taking DOACs are needed to assess whether dose adjustment to achieve therapeutic drug levels or measures of anticoagulant effect do lead to fewer thromboembolic and bleeding events. Our results indicate that these studies should include the investigation of the effect of age, as well as the established confounders of renal function, interacting drugs and body mass.

## Data Availability

Data have not been deposited or made available in any way.

## References

[1] Connolly S (2009). Dabigatran versus warfarin in patients with atrial fibrillation. N Engl J Med.

[2] Patel MR (2011). Rivaroxaban versus warfarin in nonvalvular atrial fibrillation. N Engl J Med.

[3] Granger CB (2011). Apixaban versus warfarin in patients with atrial fibrillation. N Engl J Med.

[4] Giugliano RP (2013). Edoxaban versus warfarin in patients with atrial fibrillation. N Engl J Med.

[5] Ruff CT (2014). Comparison of the efficacy and safety of new oral anticoagulants with warfarin in patients with atrial fibrillation: a meta-analysis of randomised trials. Lancet.

[6] Lippi G (2017). Direct oral anticoagulants: analysis of worldwide use and popularity using Google Trends. Ann Transl Med.

[7] Burn J, Pirmohamed M (2018). Direct oral anticoagulants versus warfarin: is new always better than the old?. Open Heart.

[8] Mitchell A (2019). Effectiveness and safety of direct oral anticoagulants versus vitamin K antagonists for people aged 75 years and over with atrial fibrillation: a systematic review and meta-analyses of observational studies. J Clin Med.

[9] Lowenstern A (2018). Interventions for preventing thromboembolic events in patients with atrial fibrillation: a systematic review. Ann Intern Med.

[10] Hellenbart EL, Faulkenberg KD, Finks SW (2017). Evaluation of bleeding in patients receiving direct oral anticoagulants. Vasc Health Risk Manag.

[11] Kirchhof P (2016). 2016 ESC Guidelines for the management of atrial fibrillation developed in collaboration with EACTS. Eur Heart J.

[12] Kailas S, Thambuluru S (2016). Efficacy and safety of direct oral anticoagulants compared to warfarin in prevention of thromboembolic events among elderly patients with atrial fibrillation. Cureus.

[13] Florey C (1993). Sample size for beginners. Br Med J.

[14] Kubitza D (2005). Safety, pharmacodynamics, and pharmacokinetics of single doses of BAY 59–7939, an oral, direct factor Xa inhibitor. Clin Pharmacol Ther.

[15] Siegal DM, Konkle BA (2014). What is the effect of rivaroxaban on routine coagulation tests?. Hematol Am Soc Hematol Educ Program.

[16] Rodgers R (2013). Correlating prothrombin time with plasma rivaroxaban level. Br J Haematol.

[17] Barrett YC, Wang Z, Knabb RM (2013). A novel prothrombin time assay for assessing the anticoagulant activity of oral factor Xa inhibitors. Clin Appl Thromb Hemost.

[18] Halperin JL (2014). Efficacy and safety of rivaroxaban compared with warfarin among elderly patients with nonvalvular atrial fibrillation in the rivaroxaban once daily, oral, direct factor xa inhibition compared with vitamin k antagonism for prevention of stroke and embolism trial in atrial fibrillation (ROCKET AF). Circulation.

[19] Garbayo JLM (2019). Hospital admissions for bleeding events associated with treatment with apixaban, dabigatran and rivaroxaban. Eur J Hospital Pharm.

[20] Eikelboom J, Merli G (2016). Bleeding with direct oral anticoagulants vs warfarin: clinical experience. Am J Med.

[21] Villines TC, Peacock WF (2016). Safety of direct oral anticoagulants: insights from postmarketing studies. Am J Med.

[22] Tamayo S (2015). Characterizing major bleeding in patients with nonvalvular atrial fibrillation: a pharmacovigilance study of 27 467 patients taking rivaroxaban. Clin Cardiol.

[23] Peacock, W.F., Patel, M., Tamayo, S., Sicignano, N., Hopf, K., Yuan, Z. (2015) Major bleeding in a post-marketing assessment of 39,052 non-valvular atrial fibrillation patients on rivaroxaban, in ESC Congress. Eur Heart J 687.

[24] Adeboyeje G (2017). Major bleeding risk during anticoagulation with warfarin, dabigatran, apixaban, or rivaroxaban in patients with nonvalvular atrial fibrillation. J Manag Care Spec Pharm.

[25] Al-Khalili F, Lindstrom C, Benson L (2016). The safety and persistence of non-vitamin-K-antagonist oral anticoagulants in atrial fibrillation patients treated in a well structured atrial fibrillation clinic. Curr Med Res Opin.

[26] Kubitza D (2013). The influence of age and gender on the pharmacokinetics and pharmacodynamics of rivaroxaban–an oral, direct Factor Xa inhibitor. J Clin Pharmacol.

[27] Sardar P (2014). Novel oral anticoagulants in patients with renal insufficiency: a meta-analysis of randomized trials. Can J Cardiol.

[28] Kubitza D (2005). Safety, pharmacodynamics, and pharmacokinetics of BAY 59–7939–an oral, direct Factor Xa inhibitor–after multiple dosing in healthy male subjects. Eur J Clin Pharmacol.

[29] Mueck W (2007). Population model of the pharmacokinetics and pharmacodynamics of rivaroxaban–an oral, direct factor xa inhibitor–in healthy subjects. Int J Clin Pharmacol Ther.

[30] Franchini M (2006). Hemostasis and aging. Crit Rev Oncol Hematol.

[31] Palta S, Saroa R, Palta A (2014). Overview of the coagulation system. Indian J Anaesthesia.

[32] Attena E. et al. (2015). Oral anticoagulation therapy in the elderly G Gerontol. 63(2).

[33] Engbers, M.J., A. van Hylckama Vlieg, and F.R. Rosendaal, Venous thrombosis in the elderly: incidence, risk factors and risk groups. J Thromb Haemost, 2010. 8(10): p. 2105-12. 10.1111/j.1538-7836.2010.03986.x 20629943

[34] Abbate R (1993). Age-related changes in the hemostatic system. Int J Clin Lab Res.

[35] Schulman S (2014). New oral anticoagulant agents—general features and outcomes in subsets of patients. Thromb Haemost.

